# Supplementary material for: Hierarchical analysis of RNA secondary structures with pseudoknots based on sections
Source: PLoS Comput Biol. 2026 Jan 27;22(1):e1013904. doi: 10.1371/journal.pcbi.1013904 (PMC12858078; doi:10.1371/journal.pcbi.1013904)
Supplement: Attachment — Submitted filename: Response to Reviewers.pdf [file pcbi.1013904.s002.pdf]

Dear Dr. Parikesit and Dr. Elofsson,

Please find attached a revised version of our manuscript (Submission ID PCOMPBIOL-D-25-01796) ***"Hierarchical Analysis of RNA Secondary Structures with Pseudoknots Based on Sections"***. We thank the editors and reviewers for the time and effort they put into reviewing the manuscript. We have carefully addressed all comments as detailed below. The major changes include:

1. Clarified worst-case complexity for 3-cluster analysis (Reviewer 1, comment 1)
2. Reported runtime and computational cost for the complete dataset (Reviewer 1, comment 3)
3. Deposited source code and datasets on GitHub and Zenodo (Reviewers 1 & 3)
4. Corrected terminology to use "nested" instead of "short-range" (Reviewer 3, comments 1 & 4)
5. Added canonical vs. non-canonical base pair analysis and clarified section definitions (Reviewer 3, comment 3)
6. Updated Table 1 with additional statistics and Figure 1 for clarity (Reviewer 1, comment 4; Reviewer 3, comments 6 & 9)
7. Incorporated pseudoknot order terminology from established literature (Reviewer 3, comment 7)
8. Demonstrated sequence length independence of prediction accuracy (Reviewer 3, comment 10)
9. Made minor revisions in the manuscript for improved clarity, precision, and readability. These revisions are reflected in both marked-up and unmarked versions of the manuscript.

We believe these revisions have significantly strengthened the manuscript and address all concerns raised. Please find our point-by-point responses (in black) to the reviewer's comments (in blue and *italics*) below.

Thank you very much.

Warmest regards,
Ee Hou Yong

1 First Report of Reviewer 1

The manuscript presents a section-based framework for RNA pseudoknot prediction. The study is clearly structured, and the proposed approach reduces computational complexity compared with conventional methods. Overall, the manuscript makes a valuable contribution to the field of RNA structure prediction. To further strengthen the quality and clarity of the work, I suggest that the authors address the following issues:

We thank the reviewer for the careful reading of our manuscript and the useful feedback.

1. *The manuscript does consider 3-cluster pseudoknots, but the authors should also provide an analysis of the worst-case scenario. While the derivation of the $O(n^2\ell^4)$ complexity is convincing, the discussion of 3-cluster cases could be expanded with a clearer comparison between the theoretical worst-case complexity (possibly $O(n^3\ell^4)$) and the practical complexity after MFE-based screening.*

   We thank the reviewer for this important question. The dominant computational cost is pairwise MFE calculation between all section pairs via dynamic programming. Each pairwise calculation requires $O(\ell^4)$ operations. With $O(n^2)$ pairs, this yields $O(n^2\ell^4)$ complexity.

   For 3-cluster analysis, we reuse these precomputed pairwise MFE values rather than computing a new joint energy function over triplets. The weighted optimization (MFE(former)$+w\times$ MFE(latter)) requires only $O(1)$ lookups per candidate triplet. Since the ℓ^4 factor is confined to the pairwise phase, total complexity remains $O(n^2\ell^4)$.

   In a hypothetical prediction scenario requiring exhaustive triplet enumeration, the enumeration cost would be $O(n^3)$ lookups with O(1) per lookup. However, the enumeration cost remains dominated by total complexity of $O(n^2\ell^4)$. The worst-case $O(n^3\ell^4)$ complexity would only arise if joint energy calculations over triplets were required, which our method avoids by design.

2. *For 3-cluster structures, the method applies different weights to the former and latter pairs (FE_former+wFE_latter). Since this algorithm is order-dependent, the authors should clarify how the order of section pairing is determined and justify the choice.*

   We thank the reviewer for pointing out that the information on section pairing order is scattered and should be described explicitly.

   The order of section pairing is determined by sequence position along the RNA in the 5' to 3' direction. Sections are numbered according to their appearance in the primary sequence. For type-1 3-clusters, the "former section pair" connects sections 1 and 2, while the "latter section pair" connects sections 2 and 3.

   This ordering is justified by the biology of RNA synthesis, which proceeds from 5' to 3'. During transcription, sections closer to the 5' end are synthesized and available for folding before downstream sections exist. This co-transcriptional folding implies that interactions between earlier sections can establish before later sections are synthesized (meyer2004co, scharfen2021transcription)

   Our results support this ordering. Former section pairs achieve prediction accuracy comparable to 2-clusters (sensitivity 0.83, PPV 0.72), while latter section pairs show lower accuracy (sensitivity 0.55, PPV 0.26). This asymmetry is consistent with hierarchical folding where earlier interactions constrain later ones, validating our choice to weight former pairs more heavily (optimal $w = 0.8$). We have added an explicit clarification in the Algorithm subsection:

   > ... *Sections are ordered by their position in the RNA sequence (5' to 3' direction), with the former section pair connecting sections appearing earlier and the latter section pair connecting sections appearing later. This ordering reflects co-transcriptional folding, where 5' regions can form interactions before 3' regions are fully synthesized.*

3. *The authors should report the actual runtime and computational cost when applying the method to the dataset of 726 tmRNA and 455 RNase P RNA sequences. This would provide a clearer picture of the practical efficiency beyond theoretical complexity.*

We thank the reviewer for this important point. We analyzed the runtime for all 1,180 sequences (726 tmRNA + 454 RNase P RNA) covering sequence lengths from 102–1,331 nucleotides.

The total execution time was 7.54 seconds, with mean runtime of 6.39 ms per sequence (median: 5.35 ms, interquartile range: 3.98–6.65 ms). The tight interquartile range demonstrates consistent performance, with only 80 sequences (6.8%) classified as statistical outliers beyond 1.5×IQR. The maximum runtime of a sequence is 63.96 ms which is highly practical. This computational efficiency enables large-scale RNA database analysis.

We report the actual runtime in our manuscript, appended to the end of the Algorithm subsection:

> *Our algorithm was implemented in C and compiled with gcc optimization flags. Runtime analysis on our complete dataset (1,180 sequences, 102–1,331 nt) showed a total execution time of 7.54 seconds, with mean runtime of 6.39 ms per sequence (median: 5.35 ms, IQR: 3.98–6.65 ms). Only 6.8% of sequences were classified as statistical outliers beyond 1.5×IQR, demonstrating consistent performance suitable for large-scale RNA database analysis.*

4. *Table 1 currently summarizes sequence characteristics. It would be helpful to also provide statistics related to pseudoknot occurrence (e.g., number of pseudoknots per sequence, proportion of 2-clusters vs. 3-clusters), which are directly relevant to the study.*

   We thank the reviewer for the valuable feedback. The reviewer is correct that Table 1 only provides summarized sequence statistics and lacks information about pseudoknots. We have revised Table 1 to include mean pseudoknots per sequence, percentage of 2-clusters, percentage of 3-clusters, and percentage of 4-clusters for TMR and RPA RNA sequences respectively.

5. *The Data and Code Availability section should include access to the test datasets used in the study.*

   We thank the reviewer for the valuable feedback. The reviewer is correct that access to the code and test datasets is critical for reproducibility, and we apologize for the omission in the initial submission. We have now uploaded all code and test datasets used in this study to Github. We have also created an archive of this repository on Zenodo.

   We have updated the "Data and Code Availability" section in the manuscript:

   > *All source code used to perform the analyses in this study, along with the complete datasets, is publicly available on Github. A permanent, citable version of the code and datasets used for this publication is archived on Zenodo.*

2 First Report of Reviewer 2

This manuscript presents a clear, well-validated hierarchical framework for pseudoknot prediction with substantial computational gains ($O(n^2\ell^4)$) and strong accuracy across large tRNA-m and RNase P datasets. The results are novel, well supported, and of broad interest to RNA structure prediction. I recommend acceptance.

We thank the reviewer for the careful reading of our manuscript, the useful feedback, and the acceptance recommendation.

3 First Report of Reviewer 3

Masuki et al. present a hierarchical method for predicting RNA structures with pseudoknots, a task often hindered by computational complexity. Their approach divides RNA into unpaired sections and predicts pseudoknot interactions between them using a nearest-neigbor energy model and dynamic programming. The method reduces computational cost while maintaining high accuracy, achieving over 0.90 sensitivity and 0.80 precision for the simplest (2-cluster) RNAs from the benchmark set. The manuscript is well-structured, the writing is coherent, clear, and mostly sufficiently detailed, although not in all parts of the text. Unfortunately, the data and software presented are not available. To ensure reproducibility and transparency, both the software implementing the proposed algorithm and the datasets used in the experiments - as well as the resulting outputs- should be made publicly available through a recognized repository such as GitHub or Zenodo. Including only code snippets or pseudocode within the manuscript is inadequate and impractical for other researchers wishing to test or build upon the presented method. If the authors choose not to release the code and data, I would strongly recommend that the manuscript be rejected, as open availability is essential for verifying the results and advancing the field. Other remarks are given below.

We thank the reviewer for the careful reading of our manuscript and the useful feedback.

1. *"RNA secondary structure is defined as the set of base pairs that form a planar graph, excluding pseudoknots which are nonplanar structural elements by definition" - this statement oversimplifies the definition of RNA secondary structure. In fact, multiple definitions exist, and a more general one describes secondary structure as simply specifying which bases are paired and which are unpaired, regardless of whether the pairings are nested or form pseudoknots (see Antczak et al., Bioinformatics 2018; doi: 10.1093/bioinformatics/btx783 - you will there a concept of hierarchical analysis and pseudoknot order that reflect the hierarchy of folding, which might be helpful in hierarchical examination MAsuki et l. use in their work). The definition adopted by the authors is a practical simplification, motivated by the difficulties associated with predicting pseudoknots and representing them in some - but not all - 2D structural notations (e.g. dot-bracket). This challenge has led to the development of methods for removing pseudoknots from 2D structures to facilitate autoated analysis (see Smit et al., RNA 2008; doi: 10.1261/rna.881308), but there remains no universal criterion for distinguishing "core" base pairs from those involved in pseudoknots (see Zok et al., AMCS 2019; doi: 10.34768/amcs-2020-0024). I suggest to clarify this conceptual nuance and reference these relevant works.*

   We thank the reviewer for pointing out that multiple definitions of RNA secondary structure exist and that our adopted definition (planar graph excluding pseudoknots) is a simplification rather than a universal standard. We have edited our Introduction to clarify this conceptual nuance supported by the instructive references provided by the reviewer:

   > *... A fundamental challenge in RNA structure prediction is defining which interactions constitute the secondary structure versus pseudoknots. RNA secondary structure has multiple definitions in literature (antczak2018new, rivas1999dynamic, brion1997hierarchy). In this work, we define RNA secondary structure as nested base pairs that can be represented without crossing lines, while pseudoknots are non-planar interactions that create crossing patterns (gray2024sparsernafold, benedetti2024rna, bon2008topological, danaee2018bprna, stadler2021alignments). While no universal criterion exists for distinguishing secondary structure from pseudoknot base pairs (smit2008knotted, zok2020new), our definition enables computational tractability,*

aligns with standard structural notations (e.g. dot-bracket format), and provides a framework for our hiearchical method of identifying unpaired sections and predicting pseudoknot interactions between them.

2. *Introduction: in the discussion of limitations and current challenges of pseudoknot prediction, I recommend referring to the work by Justyna et al. (2023, Briefings in Bioinformatics, doi: 10.1093/bib/bbad153), in which the authors benchmarked machine learning methods on experimental data, highlighting significant difficulties in accurately predicting pseudoknots compared to the prediction of canonical and non-canonical base pairs.*

   We thank the reviewer for this valuable suggestion. We have cited Justyna et al. (2023) in the Introduction section:

   > *Alternative approaches have emerged, including stochastic methods and novel computational strategies. While these techniques show promise in specific scenarios, comparative analyses reveal accuracy limitations, particularly when dealing with complex pseudoknot structures (justyna2023machine).*

3. *The manuscript would benefit from a clearer discussion of whether pseudoknots in the analyzed RNA structures are formed primarily by canonical base pairs or if non-canonical interactions also play a role. This distinction is important, as numerous studies have demonstrated that non-canonical base pairs frequently occur within pseudoknot regions and significantly affect RNA folding, stability, and tertiary interactions. Clarifying which types of base pairings are considered in the hierarchical model would deepen the reader's understanding of the method's applicability and limitations.*

   We thank the reviewer for this important question regarding the base pairs considered in our model.

   Our algorithm only considers Watson-Crick pairs (A-U, G-C) and wobble pairs (G-U). Our consideration is due to the available thermodynamic parameters in mfold 3.6 energy model, which provides experimentally-derived stacking, loop, and mismatch energies for canonical base pairs.

   The reviewer is correct that limiting our model to only canonical pairs could be a problem if pseudoknots were predominantly formed by non-canonical interactions. To address this concern, we analyzed the base pairs of all RNA structures used in our study (726 tmRNA transfer messenger RNA + 455 RNase P Ribonuclease P RNA).

   For the tmRNA dataset, we found 36,357 pseudoknot pairs, out of which 36,350 (99.98%) are canonical pairs (A-U, G-C, G-U) while 7 (0.02%) are non-canonical pairs. For the RNase P dataset, we found 10,682 pseudoknot pairs, out of which 9,688 (90.7%) are canonical pairs while 994 (9.3%) are non-canonical pairs. Thus, pseudoknots in our dataset are primarily formed by canonical base pairs. Furthermore, the high predictive accuracy our method achieved for 2-clusters (sensitivity > 0.90, PPV > 0.80) validates this modeling approach for these specific RNA classes.

   Nonetheless, we recognize that non-canonical base pairs frequently occur within pseudoknot regions and affect RNA folding, stability, and tertiary interactions. We have edited the Methods section, in particular the Algorithms subsection:

   > *... Base pairs are formed only between complementary bases (AU, UA, CG, GC, GU, UG). This restriction to canonical pairs (Watson-Crick and wobble) is due to*

the availability of thermodynamic parameters in mfold 3.6. Analysis of our datasets shows canonical pairs constitute over 99% of pseudoknot pairs in transfer messenger RNA and over 90% in ribonuclease P RNA.

4. *The use of the term "short-range" is misleading and inconsistent with standard RNA structural terminology. In RNA structure research, short-range interactions are conventionally understood to mean base-pair interactions between nucleotides that are close in the primary sequence, not those that are topologically non-crossing. I strongly suggest to adopt the established term "nested interactions" to describe non-crossing base-pair arrangements, as it is both precise and widely used in the RNA structure-related literature. Reframing the terminology would avoid confusion and better align the manuscript with accepted definitions used across computational and structural RNA studies.*

   We thank the reviewer for pointing out the incorrect usage of the "short-range" terminology. We have made the necessary edits and replaced "short-range" with "nested". We have also removed the incorrect definition of "short-range" in our manuscript. We have also taken the liberty to remove the incorrect definition of "long-range".

5. *"Common secondary structure motif includes hairpin loops, hairpin stems, helical duplexes, bulges, internal loops, multi-loops, and junctions" - What is the difference between multi-loop and junction? In the literature these terms mean exactly the same thing. Moreover, multi-loops/junctions are a subclass of internal loops. I suggest defining what exactly the authors mean by these terms.*

   We thank the reviewer for this clarification. The reviewer is correct that multi-loop and junction mean the exact same thing. We have removed the redundant term "junctions" from the sentence. However, we respectfully note that multi-loops/junctions and internal loops are considered distinct categories (Mathews et al., Journal of Molecular Biology 1999, doi: 10.1006/jmbi.1999.2700; Antczak et al., Bioinformatics 2018, doi: 10.1093/bioinformatics/btx783).

   In Mathews et al., different thermodynamic parameters and models are assigned to internal loops versus multibranch loops (which we assume to be a formal way to refer to multi-loops). Multibranch loops include a unique parameter h, representing the number of branching helices. Multibranch loops are specifically defined by $h \geq 3$ converging at a junction point, whereas internal loops connect exactly two helicases. Antczak et al. states that "diverse secondary structure motifs like hairpin apical loops, bulges, internal and n-way junction loops, separated by stems", which suggests that internal loops and multi-loops are distinct.

   To address the reviewer's concern about motif clarity, we have edited the following definition in our manuscript:

   > *Common secondary structure motif includes hairpin loops, hairpin stems, helical duplexes, bulges, internal, and multi-loops. Internal loops consist of unpaired nucleotides connecting exactly two helical segments ($h = 2$), whereas multibranch loops are junction points where three or more helices converge ($h \geq 3$).*

6. *Figure 1b:*
   - The red arrow in arc diagram obscures the fragment it points to; it needs to be positioned differently.
   - I suggest coloring sections and non-section in both representations
   - In the stem-loop representation sections are represented by blue circles - does it mean that a

section is not just a single stranded fragment but also base pairs that close a loop? If so, this is not consistent with the previously given definition of a section.
- "Linear" → "linear"
- The authors provide all 3-cluster models, I suggest adding a representation of a 2-cluster pseudoknot as well to complete the view of what they analyse in the paper. I also suggest to separate this figure from panels (a)-(b).

We thank the reviewer for these detailed suggestions. We have made the following changes to Figure 1:

1. We repositioned the red arrow to clearly indicate the non-section region without obscuring the structure.
2. We have added color coding in both representations: blue indicates sections (unpaired bases that may form pseudoknots), black indicates non-sections (bases involved in secondary structure base pairs), yellow bands represent secondary structure base pairs, and red bands represent pseudoknot base pairs.
3. We clarify that sections consist exclusively of unpaired nucleotides. In the stem-loop representation, blue circles indicate single-stranded loop regions, not base pairs. We have revised Figure 1 to make this explicit.
4. Corrected "Linear" to "linear" in the figure.
5. We have added a representative 2-cluster pseudoknot structure to panel (c) to complement the 3-cluster examples.

We respectfully retain the current figure organization with panels (a)-(c) in a single figure. Panels (a) and (b) establish fundamental definitions and terminology (secondary structure, pseudoknots, sections), while panel (c) builds on these concepts to show cluster classifications. Separating these would require readers to cross-reference between figures, reducing clarity. The unified presentation allows readers to understand the complete analytical framework in one integrated view.

7. *The complexity of a pseudoknot can be reflected by a pseudoknot order. I suggest that the authors get familiar with this concept (see my comment number 1).*

We thank the reviewer for directing us to the concept of pseudoknot order. We have incorporated this established framework in our manuscript. We begin with an addition in our methods section:

> *4.* **Pseudoknot order** *is a metric for RNA structural complexity defined by the minimum number of base pair decompositions to create a nested structure (antczak2018new). In our work, 2-clusters correspond to pseudoknot order 1 (1 decomposition; e.g. H/K-type) while 3 clusters correspond to pseudoknot order 2 (2 decompositions; L/M-type).*

and in our results section:

> *... Structures with pseudoknot order 1(2-clusters: 2337 in TMR, 543 in RPA) substantially outnumber structures with pseudoknot order 2 (3-clusters: 110 in TMR, 1 in RPA). This distribution is consistent with hiearchical folding, where lower-order pseudoknots form before higher-order structures.*

with a discussion point referencing pseudoknot order:

> ... *This sequential dependency within 3-clusters suggests that even within higher-order pseudoknots, simpler interactions must establish before complex interactions can form.*

8. *"Moreover, two sections from the same loop is prohibited because the base pairs between these sections will be in secondary structures, rather than pseudoknots." - unclear sentence, reformulate... sections will be in nested structure rather than pseudoknotted...*

   We thank the reviewer for identifying this unclear sentence. We have reformulated the unclear sentence as:

   > *Moreover, pairing between two sections from the same loop is prohibited because such base pairs would be nested rather than pseudoknotted.*

9. *Table 1:*
   - explain what do you mean by section length (see also my comment numer 6).
   - provide the information about the range of sequence length for both datasets; mean length is not enough in my opinion; besides - median is more informative than mean

   We thank the reviewer for identifying the visual ambiguity in Figure 1b relating to the definition of sections. In this work, sections consist exclusively of contiguous unpaired nucleotides. Thus, section length refers to the count of these unpaired bases. Sections do not include any base pairs. We have updated Table 1 caption to explicitly state the definition of section length:

   > ... *Section length refers to the number of unpaired nucleotides within each contiguous unpaired region.*

   We also agree that additional statistics regarding sequences used in this study should be provided in Table 1. We have revised Table 1 to include median sequence length, interquartile ranges (Q1–Q3) showing the 25th–75th percentile spread, median sections per sequence, and median section length for both TMR and RPA datasets.

10. *I suppose that the accuracy of prediction is independent on the RNA sequence length. However, it should be clearly stated in the paper (or maybe I missed it?), as this is - if I am right - quite an important feature of the method.*

    We thank the reviewer for the important observation. We performed an analysis across the sequences in our dataset (ranging from 102 to 1,331 nucleotides) to determine if the prediction accuracy depends on RNA sequence length. For each sequence, we compared predicted connections (negative MFE) against actual connections in the known structures. We calculated the true positive rate (TPR) and positive predictive value (PPV) for each sequence and examined their correlation with sequence length using Pearson correlation (r) analysis.

    For TPR vs sequence length, $r = -0.026$ ($p = 0.392$) while for PPV vs sequence length, $r = 0.046$ ($p = 0.129$). Both metrics show negligible correlation with sequence length ($|r| < 0.05$) with high p-values ($p > 0.05$), confirming no significant dependency. Under the null hypothesis of no correlation, these results support the conclusion that prediction accuracy remains consistent across the tested sequence length range.

    We have added this analysis to the manuscript at the end of the section "Comparison between MFE Structures and Real Structures for Connected Section Pairs":

To evaluate whether our method's accuracy depends on sequence length, we analyzed all RNA sequences from our dataset. For each sequence, we compared predicted connections (negative MFE values) against actual connections in known structures. We calculated the true positive rate (TPR) and positive predictive value (PPV) for each sequence and examined their correlation with sequence length using Pearson correlation analysis.

For TPR versus sequence length, $r = -0.026$ ($p = 0.392$)*, while for PPV versus sequence length,* $r = 0.046$ ($p = 0.129$)*. Both metrics show negligible correlation with sequence length* ($|r| < 0.05$) *with high p-values* ($p > 0.05$)*, confirming no significant dependency. Under the null hypothesis of no correlation, these results support the conclusion that prediction accuracy remains consistent across the tested sequence length range.*

The prediction accuracy independence is due to our hierarchical approach. Our algorithm calculates minimum free energy locally between section pairs rather than globally across the entire RNA structure. Consequently, prediction accuracy is determined by local section-pair interactions and section length ℓ *rather than total RNA length (L), consistent with our* $O(n^2\ell^4)$ *computational complexity. For a given section length, computational cost scales with the number of section pairs, not with total sequence length, ensuring that prediction accuracy remains stable regardless of overall RNA size.*

11. *(1) "involved in secondary structure base pairing" → involved in base pairing*
(2) Standardise the spelling: j-th or jth (but not both versions)
(3) $j < j'$ *We set* $C(i, j) \to j < j'$*. We set* $C(i, j)$
(4) Sensitivity is also called the true positive rate (TPR). I suggest using "TPR" in formula (8)
(5) Table 2, Table 3 - insert spaces before brackets; 1605→1,605 etc

We thank the reviewer for pointing out the minor typographical errors. Corrections have been made and are reflected in the revised manuscript.
