# Supplementary material for: Hierarchical analysis of RNA secondary structures with pseudoknots based on sections
Source: PLoS Comput Biol. 2026 Jan 27;22(1):e1013904. doi: 10.1371/journal.pcbi.1013904 (PMC12858078; doi:10.1371/journal.pcbi.1013904)
Supplement: Attachment — Submitted filename: Reviewer comments - accepted.docx [file pcbi.1013904.s001.docx]

This manuscript presents a clear, well-validated hierarchical framework for pseudoknot prediction with substantial computational gains (O(n²ℓ⁴)) and strong accuracy across large tRNA-m and RNase P datasets. The results are novel, well supported, and of broad interest to RNA structure prediction. I recommend acceptance.
